# Double Anterior Interventricular Arteries: Prevalence and Morphological Types—A Dissection Study

**DOI:** 10.3390/jpm14091007

**Published:** 2024-09-22

**Authors:** Ecaterina Daescu, Alexandra Enache, Emanuela Stan, Sorin Lucian Bolintineanu, Laura Andreea Ghenciu, Alexandra Corina Faur, Agneta Maria Pusztai, Delia Elena Zahoi

**Affiliations:** 1 Department I of Anatomy and Embryology, “Victor Babes” University of Medicine and Pharmacy, 300041 Timisoara, Romania; daescu.ecaterina@umft.ro (E.D.); s.bolintineanu@umft.ro (S.L.B.); faur.alexandra@umft.ro (A.C.F.); dzahoi@umft.ro (D.E.Z.); 2 Institute of Legal Medicine Timisoara, 300610 Timisoara, Romania; enache.alexandra@umft.ro (A.E.); emanuela.stan@umft.ro (E.S.); 3 Department of Neuroscience, Discipline of Forensic Medicine, Bioethics, Deontology and Medical Law, “Victor Babes” University of Medicine and Pharmacy, 300041 Timisoara, Romania; 4 Ethics and Human Identification Research Center, Department of Neurosciences, “Victor Babes” University of Medicine and Pharmacy, 300041 Timisoara, Romania; 5 Department of Functional Sciences, “Victor Babes” University of Medicine and Pharmacy Timisoara, 300041 Timisoara, Romania; bolintineanu.laura@umft.ro

**Keywords:** coronary arteries, morphological variations, double anterior interventricular arteries, myocardial bridges, dissection

## Abstract

Background: This study aimed to evaluate the prevalence of double anterior interventricular artery using the dissection method. Metode: A retrospective study was conducted between 2010 and 2024 at the Anatomy and Embryology Laboratory of the Victor Babes University of Medicine and Pharmacy in Timisoara. Eighty cases were analyzed for morphological variants of the coronary arteries, especially the anterior interventricular artery. Results: Two cases of double anterior interventricular arteries were identified. In the first case, the two anterior interventricular arteries originated from the anterior interventricular branch of the left coronary artery. In the second case, an additional anterior interventricular artery with an aortic origin was found running along the lower third of the two interventricular grooves. This shape has not been described before in the specialized literature. Conclusions: Knowing the potential variations of the double left anterior descending artery is critical for interpreting cardiac imaging and choosing and planning percutaneous and surgical reperfusion strategies.

## 1. Introduction 

The arterial supply of the heart is provided by the two (right and left) coronary arteries. The right coronary artery arises from the right coronary aortic sinus, with the origin typically located superior to the right semilunar valve (in 10 percent of cases, the ostium is below the cusps margin). It initially passes anterior and to the right, between the right auricle and the pulmonary trunk. Further, it reaches the atrioventricular sulcus and descends almost vertically along it to the right cardiac border, curving around it into the posterior part, where it approaches its junction with both the interatrial and interventricular sulci. The right coronary artery supplies the right ventricle, the right atrium, the sinoatrial node, the atrioventricular node, and the posterior third of the interventricular septum through its branches, with the left coronary artery supplying the apex. The left coronary artery is larger in caliber than the right and supplies a greater volume of myocardium. It originates from the left coronary aortic sinus; the ostium is below the cusp margin, leading into two major initial branches—the circumflex and anterior interventricular branches [1]. Through its branches, it supplies the left atrium, the left ventricle, and the anterior two thirds of the interventricular septum. 

The coronary arteries have numerous anatomical variations, including the site of origin, number, course, dimensions (caliber or length), branching pattern, and vascular territory, among others. Most anatomical variants have no repercussions and are discovered accidentally. However, some can lead to severe arrhythmias, the interruption of myocardial perfusion (either intermittent or chronic), or even sudden cardiac death [2]. The double anterior interventricular artery is a very rare coronary anomaly that occurs, according to various studies, with a frequency ranging from 0.13 percent to 5.96 percent of the population [3,4].

This study aimed to evaluate the prevalence of double anterior interventricular artery using the dissection method.

## 2. Materials and Methods

This study was conducted at the Department of Anatomy and Embryology of the ‘Victor Babes’ University of Medicine and Pharmacy in Timisoara, between 2010 and 2024, on a set of 80 hearts from formalin-fixed adult human cadavers, collected and dissected in the laboratory, in accordance with current legislation. Gross dissection was performed using the standard technique. We removed the pericardium and carefully examined the vascular structures: aorta, pulmonary artery, pulmonary veins, superior vena cava, and inferior vena cava were then sectioned and the heart was removed. In particular, the coronary arteries were carefully dissected. The study classified any deviations from the typical structure into distinct morphological types and analyzed numerous aspects of the coronary arteries, including their origin, number, course, and branching pattern. 

## 3. Results

The analysis of the morphological variations of the anterior interventricular artery on the study material revealed the presence of double anterior interventricular arteries in two cases (2.5 percent). 

**Figure 1:**
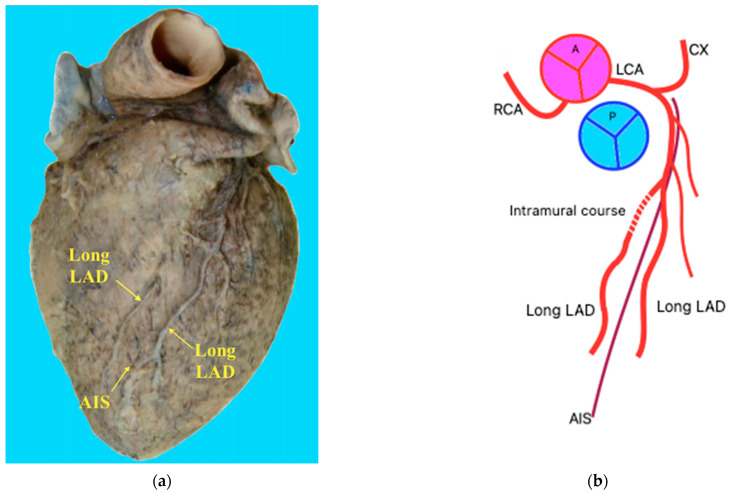
(**a**) The double anterior interventricular artery (descending anterior artery) originating from the left coronary artery, and after a short path, it branches into two long anterior interventricular arteries that descend on one side and the other of the anterior interventricular sulcus until close to the top of the heart; the right bifurcation branch presents, in its first portion, an intramuscular route. (**b**) Schematic representation.

In the first case (Figure 1), the anterior interventricular branch (anterior descending artery) originated from the left coronary artery. After a short path along the anterior interventricular groove, it branched into two long anterior interventricular arteries that descended on either side of the anterior interventricular groove, almost to the apex of the heart. Aside from the right branch’s initial intramural course, this morphological type resembles type XIII, as described by Pellegrini, J.R., et al. [5].

**Figure 2:**
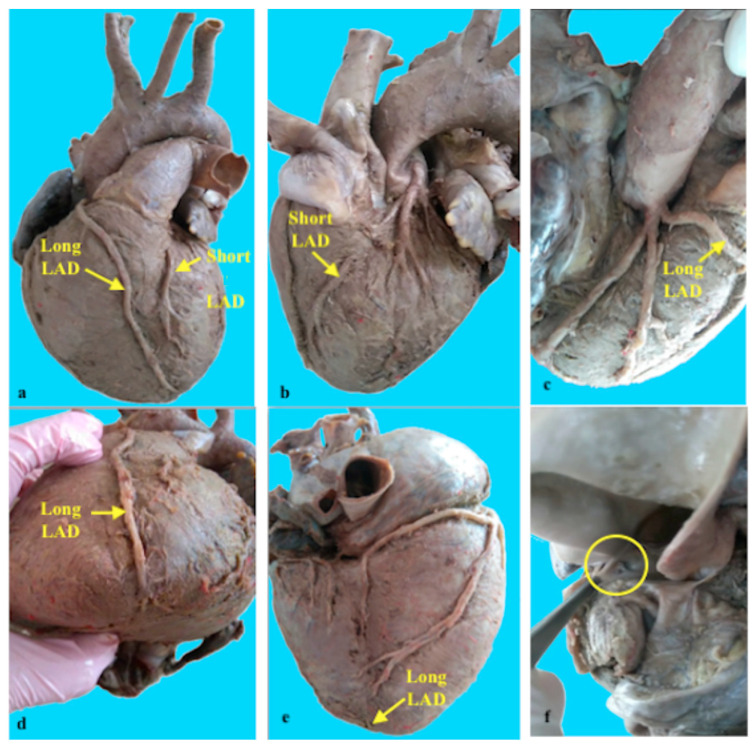
Type XIV of double anterior interventricular artery (descending anterior artery). The right anterior interventricular artery/long anterior descending artery, with a separate origin, in the right coronary sinus (**c**,**f**), which exceeds the apex of the heart approaching the distal portion of the posterior interventricular sulcus (**a**,**d**,**e**). The left anterior interventricular artery/short anterior descending artery, with its origin in the left coronary artery, presents a muscular bridge in its middle portion (**a**,**b**).

**Figure 3:**
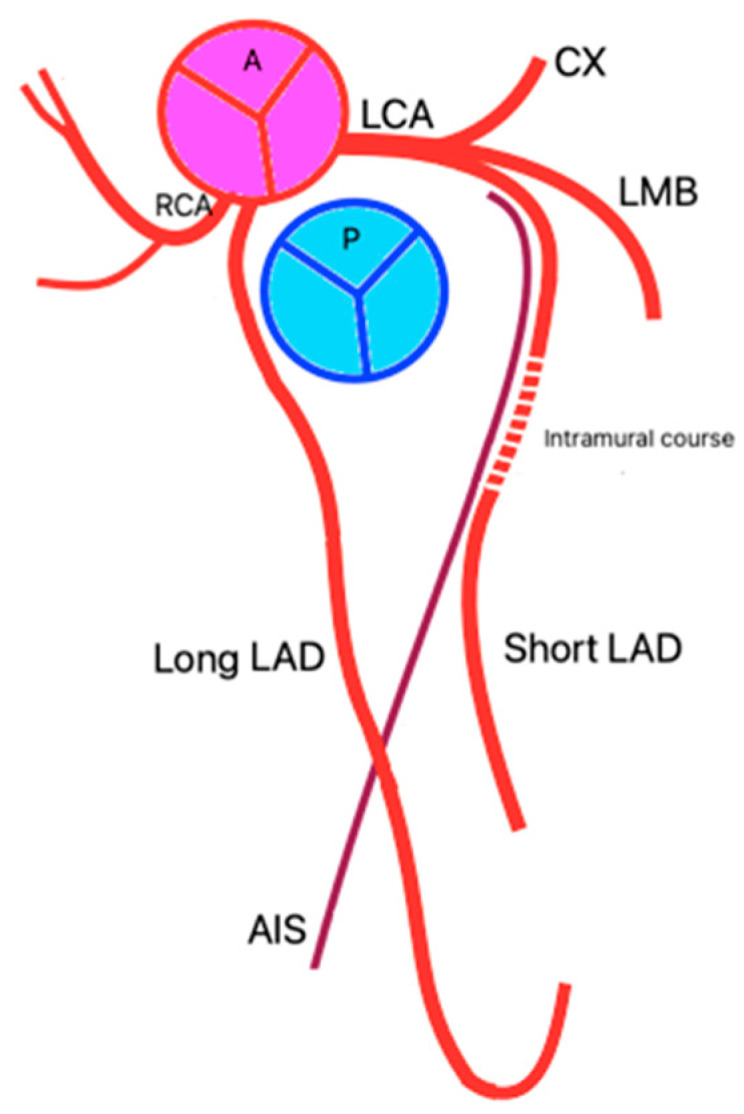
Type XIV—schematic representation.

In the second case (Figure 2 and Figure 3), an initial analysis of the origin of the coronary arteries from the aortic sinus revealed the presence of an additional artery. Starting from their aortic origins, the three arteries were dissected, and their course was traced to identify them. The right coronary artery, which arose from the right coronary sinus, was located in the coronary sulcus between the right atrium and the right auricle, extending in a posterior direction. After the right marginal branch stemmed from it, the right coronary artery continued in a posterior direction, splitting into two branches of similar size. The first branch descended diagonally down the diaphragmatic surface of the right ventricle, terminating in two branches intended for this specific region. The second branch crossed the posterior portion of the coronary sulcus to the junction with the posterior interventricular sulcus, after which it descended into the posterior interventricular sulcus, extending approximately to the middle third of this sulcus. 

The left coronary artery also exhibited morphological peculiarities. It divided into three branches of approximately equal caliber immediately after its origin: the circumflex branch, the left marginal branch, and the anterior interventricular branch. The circumflex branch traversed the posterior portion of the coronary sulcus, approached the posterior interventricular sulcus, and ended by giving off two branches destined for the diaphragmatic surface of the left ventricle. The left marginal branch followed a descending trajectory, branching out at the level of the left pulmonary surface. From its origin, the left anterior interventricular branch (anterior descending artery) positioned itself between the pulmonary artery and the left auricle, crossing the anterior portion of the coronary sulcus to reach the sternocostal surface of the heart. Shorter than usual, it traversed the upper two-thirds of the anterior interventricular sulcus, after which it exited and ended on the sternocostal surface of the left ventricle. In the middle third (between the origin and entry into the anterior interventricular sulcus), it had an intramural course (muscular bridge). The aortic sinus, anterior to the right coronary artery, was the origin of the third artery, later named the supernumerary anterior interventricular artery (right) due to its trajectory. From its origin, the artery had a short path between the right auricle (which partially covered it) and the pulmonary artery, positioning itself anterior to the latter to reach the sternocostal surface at the base of the right ventricle. From this level, the artery’s course obliquely descended toward the left. In the middle third of the sternocostal surface, the right anterior interventricular artery had a trajectory almost parallel to the anterior interventricular branch of the left coronary artery. Further, it continued its oblique descending path toward the anterior interventricular sulcus, traversing its lower third and reaching the level of the cardiac apex. After crossing the apical notch of the heart, it continued its course on the diaphragmatic surface, traversing the lower third of the posterior interventricular sulcus.

In this case, both the anterior interventricular branch (originating from the left coronary artery) and the posterior interventricular branch (originating from the right coronary artery) are present. A particular feature of this case is the presence of a long supernumerary anterior interventricular artery (right), which originates directly from the right coronary sinus and extends beyond the apical notch of the heart, entering the lower third of the posterior interventricular sulcus. The short left anterior interventricular branch (anterior descending artery) also passes through muscle in its middle third before it enters the anterior interventricular sulcus.

As a similarity, both the anterior and posterior interventricular sulci each contain two arteries. The posterior interventricular branches originating from the left coronary artery and the posterior interventricular branches originating from the right coronary artery have shorter paths; each traverses the upper two-thirds of their respective interventricular sulcus. Simultaneously, the supernumerary/right anterior interventricular artery of aortic origin traverses the lower third of both interventricular sulci.

The presented case is quite complex as it includes multiple anomalies in the number, origin, course, and termination of the coronary arteries.

## 4. Discussion

Over time, multiple studies have been conducted regarding the morphological variability of the coronary arteries, and the frequency of cases reported in the specialized literature varies depending on the size of the study sample, the method (dissection, angiography, angio-CT, or MRI), sex, geographical area, etc. Given their very low frequency, most are case presentations. 

Coronary artery anomalies correspond to patterns of heart vascularization that are generally encountered very rarely; characterized by abnormal origin, course or termination of the coronary arteries; and most of them are asymptomatic and discovered incidentally [6,7]. In the general population, they appear with a frequency ranging from 0.3 percent to 5.8 percent [2,8]. 

Andishmand, A., et al., reported an incidence of coronary artery anomalies of 1.26 percent [9]. Al-Umairi. R.S., et al. in a cohort of 4445 patients who underwent coronary-computed tomography angiography, identified 59 patients (1.3 percent) with coronary artery anomalies [10]. Kashyap, J.R., et al. reported an incidence of coronary artery anomalies of 2.06 percent [11]. Gräni, C., et al. reported an incidence of coronary artery anomalies of 2.6 percent [12]. Şahin, T., and Ilgar, M., in a study of 5200 multidetector computed tomography coronary angiography, reported a 2.61 percent incidence of coronary artery anomalies [13]. Sidhu et al. reported an incidence of coronary artery anomalies of 3.06 percent [14]. 

The anterior interventricular branch (anterior descending artery) usually originates from the left coronary artery and traverses the anterior interventricular sulcus toward the cardiac apex. Along its trajectory, it gives off several branches: the anterior conal artery, diagonal branches, and septal branches.

The double anterior interventricular artery is a rare congenital anomaly defined as the presence of two distinct arteries that approach the anterior interventricular sulcus. This category also includes cases where there are two branches, typically one short and one long, that traverse and supply different parts of the anterior interventricular sulcus; these may have different origins and courses. 

Waterson et al. made the first mention of this congenital anomaly in 1939 [15].

Double anterior interventricular arteries are very rare coronary anomalies; their reported incidence varies between 0.11 percent [10], 0.13 percent [3], 0.56 percent [11], 0.68 percent [14], 0.77 percent [13], 1.3 percent [16], and 4 percent [17].

A double left anterior descending artery arising from the left and right coronary arteries is an extremely rare congenital coronary anomaly. The specialized literature has identified several case presentations with double anterior interventricular arteries originating from different sources: the right coronary artery and the left coronary artery [3,17,18]; double anterior interventricular arteries originating from the right coronary sinus or the left coronary sinus [19]; double anterior interventricular arteries originating from the right coronary sinus or the left coronary artery [20]; and double anterior interventricular arteries originating from the right coronary sinus [21].

Spindola-Franco et al. conducted the first study that analyzed and classified double anterior interventricular arteries in 1983. They categorized these cases into four morphological types, considering the origin, course, and length of the arteries that approach the anterior interventricular sulcus [3]. Over time, researchers included additional morphological types to this primary classification (Table 1).

This study’s first case of double AIA/LAD is similar to Type XIII described by Pellegrini, J.R., et al. [5]. However, in its initial phase, the bifurcating branch that descends to the right of the AIS has an intramural course. We propose that this morphological type of double anterior interventricular artery is considered subtype XIIIA.

The second identified case has a right AIA (the long AIA/LAD), which emerges from the right coronary sinus and has a distinct ostium. It has a pre-pulmonary trajectory, moving towards the AIS and traversing its lower third to reach the cardiac apex. It then crosses the heart’s apical notch and continues its course on the diaphragmatic surface, extending through the lower third of the posterior interventricular sulcus. The left anterior interventricular artery (the short AIA/LAD) traverses the upper two-thirds of the AIS, after which it exits and terminates on the sternocostal surface of the left ventricle. In its middle third (between its origin and entry into the AIS), it exhibits an intramural course (muscular bridge).

There are no clinical data regarding any potential cardiac conditions in the individuals involved.

Therefore, we suggest classifying this case as Type XIV of the double anterior interventricular artery due to its unique characteristics.

A comprehensive understanding of these morphological variants is crucial to avoid the misinterpretation of CT coronary angiography images, as well as for accurate diagnosis, planning, and therapeutic management [23]. 

The occurrence of various coronary anomalies can be understood or explained from the perspective of the embryological development of the coronary system of the heart. 

Recent studies on embryos have clarified many aspects, but controversies still exist regarding the origin of various precursors and the mechanisms involved in the formation of the coronary system. Proepicardial cells, which cover the transverse septum and the venous sinus at the beginning of embryonic heart development, represent a major source of precursors. These cells later migrate to form the epicardium, which surrounds the heart [24]. The primordial coronary vessels appear in the 28-day-old embryo as small vascular islands formed from epicardial cells. They are located in the coronary and interventricular sulci, at the level of the cardiac apex, and in other regions of the ventricular wall [25]. At 33 days, these islands start to grow and spread out, creating multiple vascular networks in different subepicardial areas. From these structures branches penetrate deeper into the myocardium, forming small vessels and capillaries [26]. A peritruncal ring forms from the subepicardial vascular structure surrounding the initial portion of the major arteries [27,28]. Recent studies confirm that the proximal portion of the coronary arteries forms through the aortic wall penetration by the endothelial cells of the peritruncal ring, thus forming the coronary ostia [26,29]. 

Aortic wall penetration is a process of localized apoptosis in areas rich in VEGF-C receptors. The epicardium is an important place to find cells that are high in VEGF receptors, which help the coronary plexus grow [30]. Researchers have also demonstrated that the absence of VEGF inhibits the formation of ostia, alters the connection of coronary arteries with the aorta, and can thereby lead to coronary anomalies [29,31]. 

The final morphology of the coronary system depends on the branching of coronary arteries from the aorta. Any anomalies in the development or connection of the two can lead to different variants in the number or location of the coronary ostia.

## 5. Conclusions

Our study highlighted two cases of double AIA/LAD out of the eighty analyzed (2.5%), and we propose their inclusion in types XIIIA and XIV

Knowing the potential variations of the double left anterior descending artery is critical in interpreting cardiac imaging as well as in choosing and planning percutaneous and surgical reperfusion strategies. 

## Figures and Tables

**Table 1:** Classification of dual AIA/LAD.

Type	Origin Short AIA/LAD	Origin Long AIA/LAD	Course Short AIA/LAD	Course Long AIA/LAD
I [3]	AIB/LAD	AIB/LAD	Proximal AIS	Courses along the left ventricular side of the proximal AIS and re-enters the distal AIS.
II [3]	AIB/LAD	AIB/LAD	Proximal AIS	Descends along the right ventricular side of the proximal AIS and re-enters the distal AIS.
III [3]	AIB/LAD	AIB/LAD	Proximal AIS	It had an intramyocardial course in the proximal portion of the septum and appears distally at the level of the AIS or terminates intramyocardially.
IV [3]	LCA	RCA	Proximal AIS	Following an anomalous pre-pulmonic trajectory anterior to the RVOT and entering the distal AIS.
V [19]	LCS	RCS	Proximal AIS	It had an intramyocardial course before reaching the distal portion of the AIS.
VI [18]	LCA	RCA	Proximal AIS	Follows a course between the RVOT and the aortic root and then enters the distal portion of the AIS.
VII [17]	AIB/LAD	AIB/LAD	Proximal AIS	Courses on the left ventricular side of the proximal AIS and then enters the distal AIS.
VIII [17]	LCA	The middle of the RCA	Proximal AIS	Traverses the diaphragmatic surface of the right ventricle and reaches the distal portion of the AIS at the apex.
IX [17]	AIB/LAD	AIB/LAD	Proximal AIS	Passing on the LV side of the mid AIS, re-entering distally into the AIS, and ending before reaching the cardiac apex.
X [20]	LCA	RCS	Proximal AIS	Courses along an anomalous pre-pulmonic course anterior to RVOT and re-enters the distal AIS.
XI [21]	RCS	RCS	Followed an intramyocardial course, through the anterior part of the interventricular septum and terminates in the proximal AIS.	Courses along an anomalous pre-pulmonic course anterior to RVOT and re-enters the distal AIS.
XII [22]	LCA (that originates from RCS)	RCS	Proximal AIS	Courses anterior to the main pulmonary artery and terminates in the distal AIS.
XIII [5]	-	AIB/LAD	-	Two long AIA/LAD which descend to the right and left sides of the AIS, extending toward the cardiac apex.
XIIIA	-	AIB/LAD	-	Two long AIA/LAD which descend to the right and left sides of the AIS extending toward the cardiac apex; the bifurcating branch that descends to the right of the AIS has an intramural course.
XIV	LCA	RCS	Traverses the upper two-thirds of the AIS and terminates on the sternocostal surface of the left ventricle. In its middle third (between its origin and entry into the AIS), it exhibits an intramural course.	It had a pre-pulmonary course, moving towards the AIS and traversing its lower third to reach the cardiac apex. It then crosses the heart’s apical notch and continues its course on the diaphragmatic surface, extending through the lower third of the posterior interventricular sulcus.

Type XIIIA and Type XIV are described in this study.

## Data Availability

The raw data supporting the conclusions of this article will be made available by the authors on request.

## Abbreviations

Left Coronary Artery	LCA
Anterior Interventricular Artery	AIA
Left Anterior Descending Artery	LAD
Anterior Interventricular Sulcus	AIS
Left Ventricle	LV
Right Ventricle	RV
Right Coronary Artery	RCA
Anterior Interventricular Branch	AIB
Right Ventricular Outflow Tract	RVOT
Left Marginal Branch	LMB
Circumflex Artery	CX
